# An Integrative Genomics Approach for the Discovery of Potential Clinically Actionable Diagnostic and Prognostic Biomarkers in Colorectal Cancer

**DOI:** 10.3390/biomedicines13071651

**Published:** 2025-07-07

**Authors:** Mark Fertel, Duaa Mohammad Alawad, Chindo Hicks

**Affiliations:** Department of Genetics and the Bioinformatics and Computational Medicine Program, School of Medicine, Louisiana State University Health Sciences Center, 533 Bolivar, New Orleans, LA 70112, USA; mferte@lsuhsc.edu (M.F.); dalaw2@lsuhsc.edu (D.M.A.)

**Keywords:** integrating gene expression, somatic mutation, data discovery, biomarkers, colorectal cancer

## Abstract

**Background:** Despite remarkable progress in clinical management of patients and intensified screening, colorectal cancer remains the second most common cause of cancer-related death in the United States. The recent surge of next generation sequencing has enabled genomic analysis of colorectal cancer genomes. However, to date, there is little information about leveraging gene expression data and integrating it with somatic mutation information to discover potential biomarkers and therapeutic targets. Here, we integrated gene expression data with somatic mutation information to discover potential diagnostic and prognostic biomarkers and molecular drivers of colorectal cancer. **Methods:** We used publicly available gene expression and somatic mutation data generated on the same patient populations from The Cancer Genome Atlas. We compared gene expression data between tumors and controls and between dead and alive. Significantly differentially expressed genes were evaluated for the presence of somatic mutations and subjected to functional enrichment analysis to discover molecular networks and signaling pathways enriched for somatic mutations. **Results:** The investigation revealed a signature of somatic mutated genes transcriptionally associated with colorectal cancer and a signature of significantly differentially expressed somatic mutated genes distinguishing dead from alive. Enrichment analysis revealed molecular networks and signaling pathways enriched for somatic mutations. **Conclusions:** Integrative bioinformatics analysis combining gene expression with somatic mutation data is a powerful approach for the discovery of potential diagnostic and prognostic biomarkers and potential drivers of colorectal cancer.

## 1. Introduction

Colorectal cancer (CRC) is the third most commonly diagnosed malignancy and the second most common cause of cancer-related death in the United States (US) [1]. According to the American Association of Cancer Research (AACR), there were an estimated 153,020 new cases of CRC and 52,550 deaths from the disease in the US in 2023 [1]. These numbers included 19,550 new cases and 3750 deaths in individuals younger than 50 years [1]. The rapidly shifting trend to diagnosis at a younger age, at a more advanced stage, and increasing incidences and mortality rates in the younger population pose major challenges in the clinical management of CRC [1]. The increases in incidence and mortality rates are particularly more alarming in developing countries with limited capacity and resources [2]. According to The Global Cancer Observatory (GLOBOCAN), there were an estimated 1.9 million new cases of CRC and over 900,000 deaths from the disease worldwide in 2022, making it the third most diagnosed cancer and the second leading cause of cancer-related deaths [3]. With the continued upward trend in the incidence of CRC in developing countries, the incidence of CRC worldwide is predicted to increase to 2.5 million new cases annually by 2035 [4] and to 3.2 million newly diagnosed cases and 1.6 million deaths annually worldwide by 2040 [4]. These alarming numbers heighten the urgent need for the discovery of biomarkers for early detection of the disease, identifying individuals at high risk of developing aggressive tumors who could be prioritized for treatment, and the discovery of targets for the development of novel, more effective therapeutics.

The recent surge of next-generation sequencing technologies has enabled the molecular classification of CRC using gene expression data [5,6]. Discoveries from these analyses have increased our understanding of the molecular taxonomy of CRC, revealing the phenotypic heterogeneity of the disease [5]. Recent large multicenter projects such as The Cancer Genome Atlas (TCGA) [6,7] and the International Cancer Genome Consortium (ICGC) [8] have performed detailed analyses of CRC genomes. Discoveries from these analyses are poised to advance precision medicine [9]. Traditionally, a majority of genomic analyses in CRC have conducted analyses on gene expression and somatic mutation as separate research endeavors [5,6]. A number of studies have reported gene expression analysis in CRC, revealing the heterogeneity of these diseases [5,6]. Similarly, a number of studies have reported analysis of somatic mutations, including analysis of copy number variation in CRC, providing valuable insights about the genomic basis of the disease [10,11,12,13].

Despite advances in genomic research, few studies have systematically combined gene expression profiles with somatic mutation data to identify diagnostic and prognostic biomarkers for colorectal cancer (CRC) within the same patient population. Integrating these two molecular layers offers a more comprehensive approach for the discovery of biomarkers and therapeutic targets with potential direct clinical relevance. Given the availability of datasets that include both transcriptomic and mutational information from the same patients, there is now a unique opportunity to leverage this integrative strategy to inform clinical decision-making to improve clinical management of the disease and outcomes in CRC patients.

The objectives of this study were to leverage gene expression data and integrate it with somatic mutation information for the discovery of potential diagnostic and prognostic biomarkers and potential targets for the development of novel therapeutics. Our working hypothesis was that significantly differentially expressed genes transcriptionally associated with CRC and genes distinguishing individuals who died (dead) from those who survived (alive) are somatically mutated. We further hypothesized that these genes are functionally related and interact with one another in gene regulatory networks and signaling pathways enriched for somatic mutations which drive CRC tumorigenesis. We addressed these hypotheses using publicly available gene expression data and somatic mutation information derived from the same individuals diagnosed with CRC from the TCGA.

## 2. Materials and Methods

### 2.1. Overall Study Design and Execution Strategy

**Figure 1:**
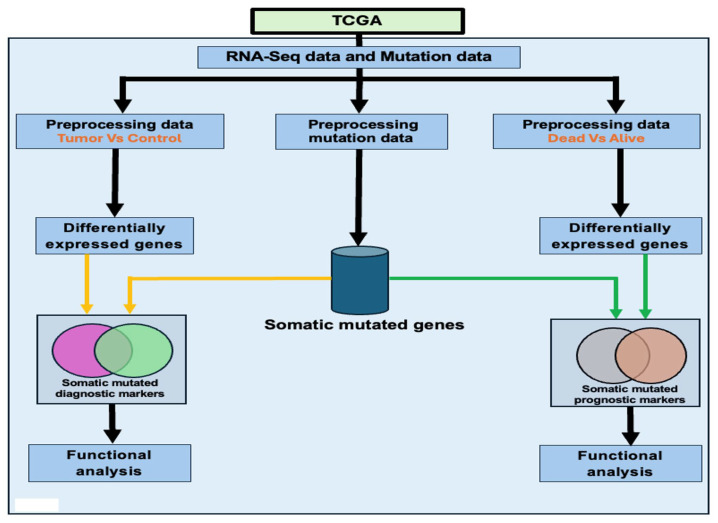
Overall study design and execution workflow. This figure depicts the steps taken in developing an integrative bioinformatics framework for integrating gene expression data with somatic mutation information to discover potential diagnostic and prognostic biomarkers and therapeutic targets. TCGA denotes The Cancer Genome Atlas, the source of gene expression and somatic mutation data used in the investigation.

A key knowledge gap and critical unmet medical need addressed in this study centers around integrating gene expression with somatic mutation data for the discovery of potential diagnostic and prognostic biomarkers and therapeutic targets. The overall study design and analysis workflow are shown in Figure 1. We used publicly available RNA-Seq and somatic mutation data on CRC generated on the same individuals from TCGA [14]. The scientific premise was that both gene expression and somatic mutation alterations contribute to the pathogenesis of CRC and that combining the two datasets would provide new knowledge for the discovery of potential diagnostic and prognostic biomarkers that could not be attained using individual datasets.

### 2.2. Sources of Gene Expression and Somatic Mutation Data

We used publicly available gene expression (RNA-Seq) and somatic mutation data, along with associated clinical information, obtained from the TCGA generated from tumor and control samples derived from individuals diagnosed with colorectal cancer (CRC) [14,15,16] (accessed on 24 October 2024). Data retrieval was performed using the Genomics Data Commons (GDC) Data Transfer Tool, which provides a secure and efficient download of large-scale genomics files [15,16]. Details regarding the cohort composition and experimental protocols are available from the original TCGA publications [14]. Below, we present a concise summary of the cohort characteristics and data types used in our analysis.

The RNA-Seq dataset included gene-level raw read counts derived from both tumor and matched normal tissue (control) samples. Tumor and control samples were initially downloaded in separate batches and subsequently merged to construct a unified data matrix, where rows corresponded to individual genes and columns to samples. Sample barcodes and identifiers were used to link the molecular data with the associated clinical metadata. The final dataset comprised N = 843 samples, including N = 790 tumor samples and N = 53 normal controls. Within the tumor group, N = 495 individuals were classified as alive and N = 127 as dead. Samples lacking defined vital status were excluded from downstream analyses. The original expression matrix consisted of 60,660 probes (transcripts), which were mapped to corresponding gene symbols for subsequent processing.

Somatic mutation data were generated using whole-exome sequencing of tumor samples, consistent with the TCGA protocol [14]. Somatic mutation data were retrieved from the Genomic Data Commons (GDC) in a Mutation Annotation Format (MAF) file. The MAF file provided comprehensive details on mutation types, somatic mutated genes, chromosome positions, mutation locations, and the corresponding samples in which mutations were detected. The dataset included 20,155 somatically mutated genes containing single-nucleotide polymorphisms (SNPs), as well as insertions (INSs) and deletions (DELs).

To enable integrative analysis, we used sample identifiers and gene symbols to align the somatic mutation data with gene expression and clinical outcome information, retaining only those samples that contained complete information across all three data types, including survival status (alive or dead). We annotated and processed somatic mutation data using the Maftools R package (version 2.24.0), a specialized tool for analyzing and visualizing mutation annotation files [17]. A file containing annotated somatic mutation (SNP, INS, and DEL) used in this study is provided in Supplementary Table S1 in this report. The original somatic mutation data file is downloadable from the GDC: https://gdc.cancer.gov/. Using the annotated data file, we further processed the Mutation Annotation Format (MAF) file and created two data matrices containing somatic mutations: a data matrix of somatic mutations on all individuals diagnosed with CRC and a data matrix of somatic mutations on dead versus alive. These data matrices were used in downstream integrative analysis to quantify the number and frequency of somatic mutations to identify significantly differentially expressed somatic mutated gene signatures distinguishing tumor from normal samples, dead from alive, and for functional enrichment analysis to identify potential therapeutic targets.

### 2.3. Bioinformatics Analysis of RNA-Seq Data

To ensure high-quality data for downstream analyses, we employed rigorous data preprocessing steps using the data processing and analysis pipeline for the RNA-Seq dataset we have developed [18]. We filtered out probes with zero and very low expression values across the samples to minimize noise and enhance the robustness of statistical analyses. Under this processing step, probes with expression values less than 10 counts across samples were excluded to ensure that only informative probes with adequate expression levels across the dataset were retained. This filtering process was applied in two stages. First, the N = 790 tumor samples and N = 53 normal samples were used for the discovery of a signature of potential diagnostic biomarkers transcriptionally associated with CRC. This filtering process reduced the number of probes from 60,660 to 26,347 informative probes, which were used in the downstream analysis. In the second step, we removed uninformative probes from the N = 495 alive versus N = 127 dead tumor samples used for the discovery of a signature of potential prognostic biomarkers distinguishing the two patient subpopulations. This filtering process reduced the number of probes from 60,660 to 21,596 probes, which were used in the downstream analysis.

Following the data filtering steps, we transformed the expression values to a Log2 to eliminate the effects of scale that could affect the comparison of gene expression levels between the tumors and control samples and between the deceased and alive patient subpopulations. Using log-transformed data, we converted probes to gene symbols using the Ensemble database using the BioMart tool [19]. We normalized the data using the median-of-ratios method implemented in DESeq2 (version 1.46.0), a software popularly used for analysis of RNA-Seq data [20]. This normalization method corrects for sequencing depth variations across samples by calculating size factors based on the median ratio of observed counts relative to a pseudo-reference sample [20]. This normalization procedure ensures that gene expression levels are directly comparable across samples, mitigating any potential biases that could be introduced by differences in sequencing depths [20]. In addition, we performed variance stabilization analysis using the variance stabilizing transformation (VST) implemented in DESeq2 to further stabilize the variance and improve data interpretability [20]. The VST method addressed the strong mean–variance relationship characteristic of RNA-Seq count data by transforming raw counts into a scale where the variance remains relatively constant across different expression levels, allowing for unbiased comparison of gene expression levels across samples and patient groups.

Using normalized datasets, we performed whole transcriptome supervised analysis comparing gene expression levels between tumor samples and controls using a generalized linear model (GLM) implemented in DESeq2 [20] to identify a signature of significantly differentially expressed genes (DEGs) between the tumor and control samples. Separately, we conducted whole-transcriptome analysis comparing gene expression values from tumor samples between dead and alive patients to identify differentially expressed genes (DEGs) distinguishing the two groups. In both analyses, we used the Wald test as implemented in DESeq to compute the *p*-values [20,21]. In addition to estimates of the *p*-values, we computed the log-fold changes (log2FC) [20]. We computed the correlation coefficients for each gene to determine whether expression values were correlated with the clinical phenotype and the direction of change in gene expression defined as up- or downregulated. In both analyses, we used the Benjamin–Hochberg false discovery rate procedure (FDR) to control for multiple hypothesis testing [22]. The genes were ranked based on the estimated *p*-values, FDR, and the log2FC.

### 2.4. Integrating Gene Expression with Somatic Mutation Information

Following unbiased whole transcriptome analyses of gene expression data, we integrated gene expression with somatic mutation data separately for each analysis. First, we evaluated the set of all significantly (*p* < 0.05) differentially expressed genes associated with CRC for the presence of somatic mutations. This was done to identify a signature of significantly differentially expressed somatic mutated genes transcriptionally associated with CRC. We quantitatively assessed the number and frequency of somatic mutations per gene in tumor samples. Genes were ranked on estimates of the *p*-values, FDR, and log2FC and number of somatic mutations. Secondly, we evaluated the set of all significantly (*p* < 0.05) differentially expressed genes distinguishing dead from alive for the presence of somatic mutations. We quantitatively assessed the number and frequency of somatic mutations per gene in dead and alive tumor samples. Thus, for deceased versus alive, the genes were ranked on estimates of the *p*-values, FDR, and log2FC and number of somatic mutations per gene within each patient group to identify differentially mutated genes between the two patient groups. In both analysis strategies, we used Volcano plots to visualize the distribution of the results of the analyses.

### 2.5. Functional and Enrichment Analysis

After identifying a set of somatically mutated genes transcriptionally associated with colorectal cancer (CRC), along with another set of significantly differentially expressed somatic mutations distinguishing deceased from surviving patients, we selected the top 500 most highly significantly differentially expressed and differentially mutated genes from each analysis. Using the top 500 most highly significantly differentially expressed and differentially mutated genes from tumors versus controls and the top 500 most highly significantly differentially expressed and differentially mutated genes from dead versus alive, we separately performed unsupervised analysis using hierarchical clustering implemented in the R package [23]. Hierarchical clustering was performed using Euclidean distance as the measure of distance between pairs of genes and complete linkage as the method of clustering [18,19,20,21,22,23]. Using the same sets of genes, we performed Gene Ontology (GO) analysis [24] using ClusterProfiler [25] and ShinyGo [26] to characterize the genes according to the biological processes, cellular components, and molecular functions in which they are involved. Subsequently, using the same sets of top genes, we performed network and pathway analyses separately using the Ingenuity Pathway Analysis (IPA) platform [27] to identify molecular networks and signaling pathways enriched for somatic mutations. For each analysis, we computed the Z-scores and *p*-values to assess the reliability of correctly predicting the molecular networks and functional category to which the genes map. We computed the log *p*-value using Fisher’s exact test as implemented in IPA to predict the signaling pathways enriched for somatic mutations.

## 3. Results

The primary objectives of this study were to leverage gene expression data and integrate it with somatic mutation information to discover potential diagnostic and prognostic biomarkers and targets for the development of novel therapeutics. We addressed these objectives in two parts using gene expression and somatic mutation data linked with clinical information generated on the same individuals. The first part of the investigation focused on leveraging gene expression data and integrating it with somatic mutation information to discover potential diagnostic biomarkers and therapeutic targets using data on tumor and control samples. The second part of the investigation focused on leveraging gene expression data and integrating it with somatic mutation information for the discovery of potential prognostic biomarkers and therapeutic targets using data from dead versus alive tumor samples. This section summarizes the results from the two investigations.

### 3.1. Discovery of a Signature of Genes Transcriptionally Associated with CRC

To address the hypothesis that there are significant differences in gene expression between tumor and normal samples, we performed a supervised analysis comparing gene expression between tumor and control samples. The goal was to identify a signature of significantly differentially expressed genes between tumor and control samples that could serve as potential diagnostic biomarkers, which could be evaluated for somatic mutations.

Analysis of gene expression differences between tumor and control samples revealed a signature of 20,122 significantly differentially expressed (*p* < 0.05) transcriptionally associated with colorectal cancer (CRC), confirming our hypothesis. Among the genes in the signature, 12,803 genes were upregulated and 7320 genes were downregulated in tumor samples compared to controls. The results showing the distribution of significantly differentially expressed up- and downregulated genes are presented in a volcano plot in Supplementary Figure S1. A complete list of all the 20,122 significantly differentially expressed genes associated with CRC, along with their estimates of *p*-values, log2FC, and correlation coefficients, is presented in Supplementary Table S2.

### 3.2. Signature of Somatic Mutated Genes Transcriptionally Associated with CRC

Having discovered a signature of genes transcriptionally associated with CRC, we now focused on addressing the objective of part 1 of the study. That is, to investigate the power of integrating gene expression data with somatic mutation information for the discovery of potential diagnostic biomarkers and therapeutic targets using data on tumor and control samples. The underlying hypothesis was that significantly differentially expressed genes transcriptionally associated with CRC are somatically mutated and are likely involved in CRC tumorigenesis. To address this hypothesis, we evaluated the 20,122 significantly differentially expressed genes transcriptionally associated with CRC for the presence of somatic mutations. The goal was to identify a signature of significantly differentially expressed and differentially somatic mutated genes transcriptionally associated with CRC. To evaluate the involvement of the identified genes in CRC tumorigenesis and to test their ability to function as potential diagnostic biomarkers, we conducted in silico validation by examining existing literature on CRC, focusing on the top most highly significantly differentially expressed somatically mutated genes.

**Figure 2:**
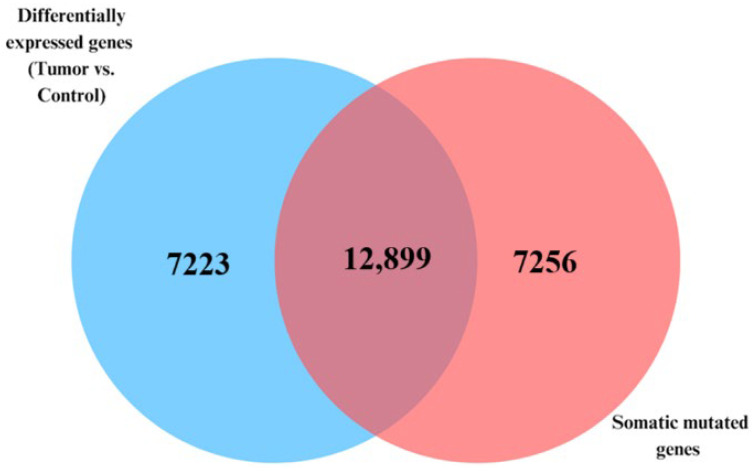
Venn diagram showing significantly (*p* < 0.05) differentially expressed somatic mutated genes (12,899 genes in intersection) in tumor versus control samples. The blue circle represents genes that were significantly differentially expressed before integration with somatic mutation information, while the red circle represents genes containing somatic mutations before integration with differentially expressed genes.

The Venn diagram in Figure 2 presents the results of an integrative analysis combining gene expression with somatic mutation. The analysis revealed a set of 12,899 genes that were both significantly differentially expressed (*p* < 0.05) between the tumor and control samples and contained somatic mutations. Notable examples within this intersecting group of genes included *CDH3*, *ETV4*, *ESM1*, and *KRT80*, all previously implicated in CRC progression and tumor microenvironment modulation. In addition, the analysis revealed a signature of 7223 genes that were differentially expressed but lacked somatic mutations. Conversely, the analysis revealed a signature of 7256 genes that were somatically mutated but not differentially expressed, including genes like *PCDHAC2*, *ZFP42*, and *SLC7A11*. This suggests that, although both gene expression and somatic alterations contribute to CRC tumorigenesis, their actions may happen at varied time points during the course of the disease. Gene expression can be time-specific, whereas somatic mutations follow an evolutionary process during the course of tumor development and progression. Under such conditions, the observed outcome is expected.

A list of the top 40 most highly significantly differentially expressed highly somatically mutated genes, along with their estimates of gene expression *p*-values and the number of somatic mutations per gene, is presented in Table 1. Also present in the table are the references from an in silico analysis supporting evidence of association with CRC, the discovered genes. As evidenced from the results in Table 1, in silico validation of the 40 highly differentially expressed highly somatic mutated genes transcriptionally associated with CRC revealed that the genes *CDH3*, *KRT80*, *ETV4*, *ESM1*, *FOXQ1*, *WNT2*, *CLDN1*, *AJUBA*, *NFE2L3*, *BEST4*, *CPNE7*, *INHBA*, *MTHFD1L*, *MMP7*, *PEX26*, *KLK6*, *TRIB3*, *KRT23*, *CEMIP*, *TRIP13*, *PHLPP2*, *SLCO4A1*, *MDFI*, *NOTUM*, *ENC1*, *VWA2*, and *LARGE2*, have been experimentally confirmed to be directly involved in CRC [28,29,30,31,32,33,34,35,36,37,38,39,40,41,42,43,44,45,46,47,48,49,50,51,52,53,54]. A comprehensive list of the 12,898 somatically mutated genes that show significant differential expression associated with CRC, including their *p*-values, log-fold changes (log2FC), and mutation counts per gene, is provided in Supplementary Table S2 of this report.

### 3.3. Discovery of Molecular Drivers of CRC as Potential Therapeutic Targets

To investigate the functional relationships among the discovered genes and to identify molecular networks and signaling pathways enriched for somatic mutations, we performed hierarchical clustering, GO, and pathway analysis using the top 500 highly significantly differentially expressed somatically mutated genes transcriptionally associated with CRC. Using GO analysis, genes were characterized according to the biological processes (BP), cellular components (CC), and molecular functions (MF) in which they are involved.

**Figure 3:**
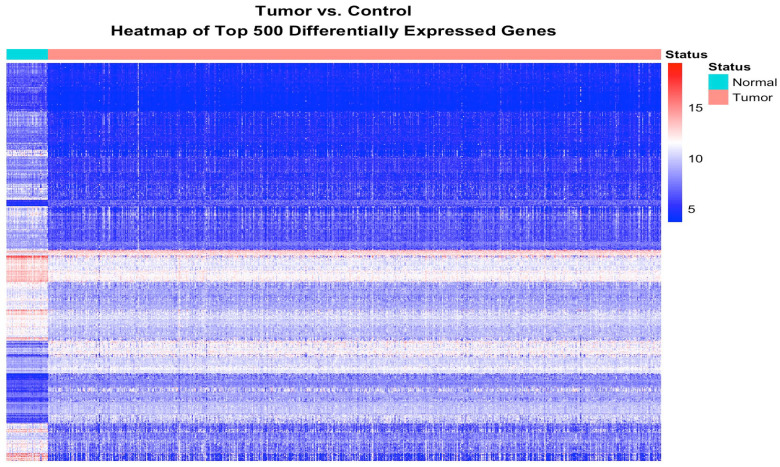
Heatmap generated from hierarchical clustering of the top 500 highly significantly differentially expressed somatic mutated genes discovered by comparing gene expression levels between the tumor and control samples. Each row corresponds to a specific gene, while each column represents an individual sample, highlighting variations in patterns of gene expression across the sample groups. The color gradient represents expression levels for downregulated (blue) and upregulated (red).

The results showing the patterns of expression profiles for the 500 most highly significantly differentially expressed somatic mutated genes transcriptionally associated with CRC are presented in Figure 3. The analysis produced clusters of genes with similar patterns of expression profiles (Figure 3), confirming our hypothesis that somatic mutated genes transcriptionally associated with CRC are co-regulated. As expected, there was significant variation in the patterns of gene expression profiles among the tumor samples (Figure 3). The observed spuriousness in the patterns of gene expression profiles can be partially explained by the genetic and phenotypic heterogeneity of CRC.

**Figure 4:**
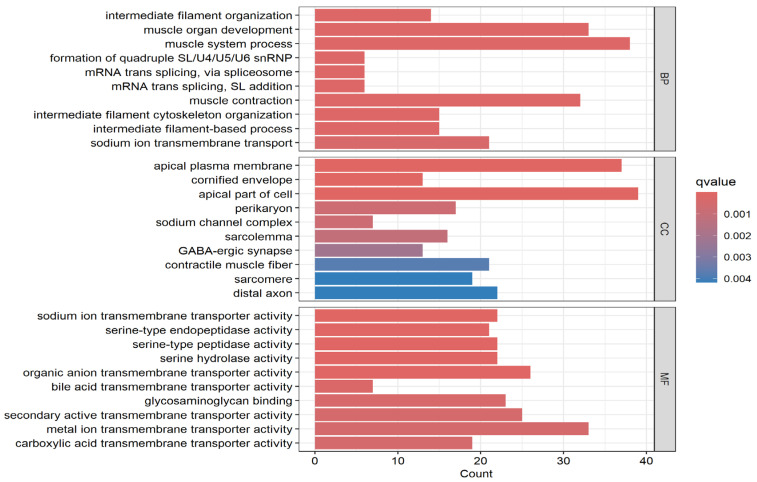
The most highly significant Gene Ontology (GO) enriched terms for the biological process (BP), cellular component (CC), and molecular functions in which significantly differentially expressed somatic mutated genes transcriptionally associated with CRC are involved. The *x*-axis represents the number of somatic mutated genes transcriptionally associated with disease mapping to the BP, CC, and MF categories. The *y*-axis shows the GO terms for BP, CC, and MF in which somatic mutated genes transcriptionally associated with CRC are involved.

The results of the functional analysis for the three GO categories are presented in Figure 4 for the most significant GO terms. Also presented in Figure 4 are the number of input genes mapped to each GO category. The analysis revealed various GO categories for molecular functions, biological processes, and cellular components in which somatic mutated genes transcriptionally associated with CRC are involved (Figure 4).

Evaluation based on the biological process in which they are involved revealed that somatically mutated genes transcriptionally associated with CRC are involved in various biological processes, including filament organization, organ development, mRNA splicing, muscle contraction, and transport (Figure 4). Evaluation of the genes based on cellular components reveals that various cellular components are involved in the genes (Figure 4). Evaluation based on molecular functions revealed involvement of the genes in various molecular functional activities, including endopeptidase, peptidase, hydrolase, and transport activities (Figure 4).

**Figure 5:**
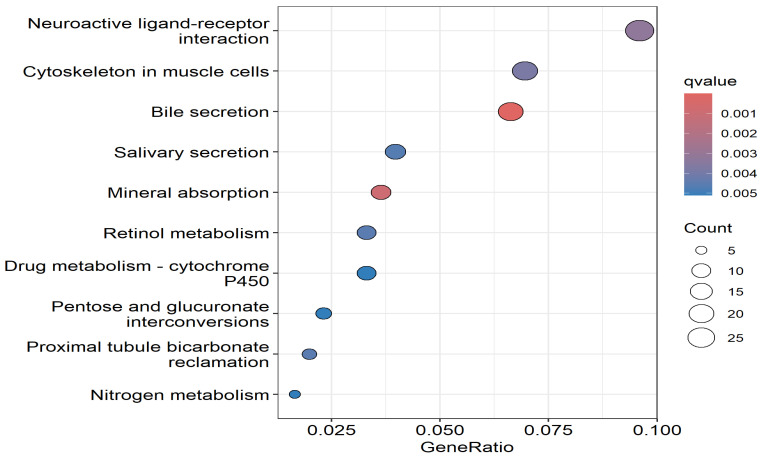
Top signaling containing somatic mutated genes transcriptionally associated with CRC discovered using enrichment analysis. The bubble plot highlights pathways significantly enriched for somatic mutated genes transcriptionally associated with CRC. The *x*-axis represents the gene ratio (proportion of genes involved in each pathway), while the color gradient indicates statistical significance based on q-values (adj *p*-values). Larger bubbles reflect a higher count of genes enriched for somatic mutations in each pathway.

The top signaling pathways enriched for somatic mutations are shown in Figure 5. Pathway analysis revealed several pathways reported to be involved in CRC, among them being cytoskeleton regulation, bile secretion, mineral absorption, retinol metabolism, drug metabolism, and nitrogen metabolism [55,56,57,58]. These results suggest that somatic mutations in CRC affect various signaling pathways.

Several genes from the enriched signaling pathways have been reported to be involved in CRC [33]. These included *WNT2*, a key driver of the Wnt/β-catenin signaling pathway, which promotes tumor cell proliferation, invasion, and metastasis [33]. *CLDN1*, involved in tight junctions and cytoskeletal organization, is overexpressed in CRC and enhances metastatic potential by disrupting cell adhesion and promoting epithelial–mesenchymal transition (EMT) [34]. *CDH3* (P-cadherin), a calcium-dependent adhesion molecule, is upregulated in CRC and contributes to reduced adhesion, increased invasiveness, and poor prognosis [28]. *EPHX4*, a metabolic enzyme associated with retinol and drug metabolism, influences detoxification and epithelial homeostasis, thereby impacting CRC development [59]. Additionally, pathways such as bile secretion, mineral absorption, nitrogen metabolism, and pentose and glucuronate interconversions reflect metabolic reprogramming, a hallmark of cancer [59]. For instance, disrupted bile acid metabolism can lead to DNA damage and inflammation, while altered mineral and nitrogen metabolism support the high energy demands of proliferating tumor cells [59]. Together, these findings highlight how *WNT2*, *CLDN1*, *CDH3*, and *EPHX4* are likely to contribute to CRC pathogenesis through interconnected mechanisms involving signaling, adhesion, and metabolic regulation.

Taken together, the results of the first part of this study show that integrative bioinformatics analysis combining gene expression data with somatic mutation information generated on the same individuals diagnosed with CRC tumors is a powerful approach for the discovery of potential diagnostic biomarkers and potential therapeutic targets.

### 3.4. Discovery of a Signature of Differentially Expressed Somatic Mutated Genes Distinguishing Dead from Alive

The second part of this study focused on leveraging gene expression data and integrating it with somatic mutation information to discover a signature of significantly differentially expressed somatic mutated genes between dead and alive. The rationale is that such genes could serve as potential prognostic biomarkers and potential therapeutic targets. Our working hypothesis was that sequential sampling and analysis comparing gene expression levels and somatic mutation frequency between dead and alive would lead to the discovery of a signature of significantly differentially expressed and somatic mutated genes. We addressed this hypothesis by comparing gene expression levels between dead and alive. Significantly differentially expressed genes from this analysis were evaluated for the number and frequency of somatic mutations within each group of patients to identify a signature of significantly differentially expressed differentially somatic mutated genes distinguishing the two patient subpopulations. The distribution of the results from this analysis is shown in a volcano plot in Supplementary Figure S2.

**Figure 6:**
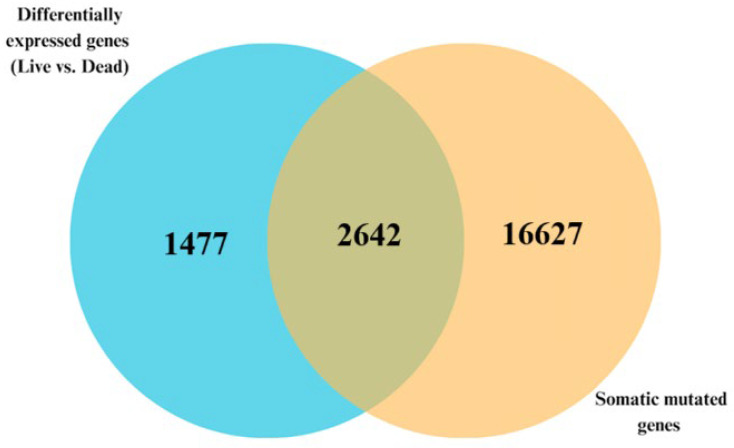
Venn diagram showing the numbers of significantly differentially expressed somatic mutated genes (2642 genes in the intersection) in alive versus deceased samples. The blue circle represents genes that were significantly differentially expressed before integration with somatic mutation information, while the gold circle represents genes that contain somatic mutations before integration with differentially expressed genes. The intersection contains genes that were both significantly differentially expressed and somatic mutated discovered following data integration.

The Venn diagram in Figure 6 presents the distribution of the genes from the analyses. Whole-transcriptomic analysis comparing gene expression levels between dead and alive revealed a signature of 4795 significantly (*p* < 0.05) differentially expressed genes. Among the genes in the signature, 2642 genes were both differentially expressed and somatically mutated, confirming our hypothesis. The list included the genes *MAD1L1*, *HECW1*, and *ANKIB1*, which have been reported to be involved in mitotic checkpoint control and have been previously implicated in tumor progression [60]. Additionally, integrative analysis identified 1477 genes that were transcriptionally altered but not somatically mutated between the two groups, including *NDUFA4P1*, *LINC02362*, and *NR2F2-AS1*. On the other hand, 16,627 genes were somatically mutated without significant differences in expression between the deceased and alive samples, including the genes *PCDHAC2*, *ZFP42*, and *SLC7A11*. The discovery of a larger number of somatically mutated genes that were more differentially expressed was expected, because although both gene expression and somatic alterations contribute to CRC tumorigenesis, their actions happen at varied time points during the course of the disease. Here, gene expression analysis was based on a cross-sectional approach of comparing expression levels between dead, whereas quantification of somatic mutations was based on the entire disease course, including the development and progression stages. Thus, to the extent that gene expression can be time-specific and somatic mutations follow an evolutionary process during the course of tumor development and progression, under such conditions, the observed outcome was expected.

To assess the differences in somatic mutations between the dead and alive, we ordered the genes based on somatic mutation count in each patient group. Table 2 shows a list of the top 40 most significantly differentially expressed and somatically mutated genes distinguishing the two groups. In silico validation of this gene list revealed the genes *TTN*, *CCDC144B*, *TDRD1*, and *ANKRD36,* which have been reported to be involved in CRC pathogenesis [59,60,61]. A complete list of all the 2642 significantly differentially expressed somatic mutated genes distinguishing dead from alive is provided in Supplementary Table S3. Analysis of the patterns of genes expression and somatic mutations in the dead versus alive samples revealed substantial variability in the expression profiles between the two patient populations. The observed variability in the patterns of gene expression and mutation profiles can be partially explained by the imbalance in sample sizes in the two patient groups and phenotypic heterogeneity of CRC.

### 3.5. Discovery of Potential Drivers of CRC and Potential Therapeutic Targets

To understand the biological mechanisms in which differentially expressed somatically mutated genes distinguish dead from alive samples and to identify the pathways enriched for somatic mutations, we performed GO and pathway analyses using the top 500 most highly significantly differentially expressed somatically mutated genes. The underlying hypothesis was that differentially expressed somatic mutated genes distinguishing dead from alive samples are functionally related and interact with one another in signaling pathways that likely drive CRC progression. We performed in silico validation on the top most highly differentially expressed differentially somatic mutated genes distinguishing the two patient subpopulations to assess the potential clinical utility of the discovered genes.

**Figure 7:**
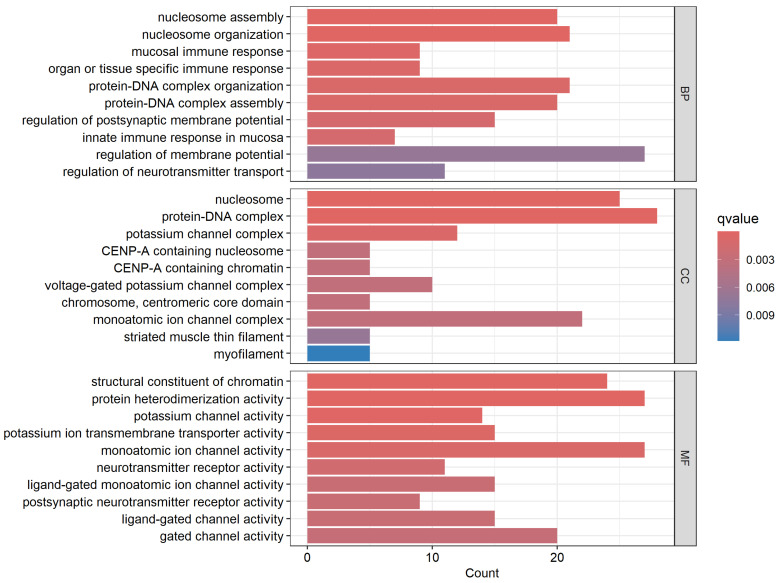
The most highly significant Gene Ontology (GO) enriched terms for the biological process (BP), cellular component (CC), and molecular functions (MF) in which significantly differentially expressed somatic mutated genes distinguishing deceased from alive are involved. The *x*-axis represents the number of significantly differentially expressed somatic mutated genes distinguishing the two patient groups mapped to the BP, CC, and MF categories. The *y*-axis shows the names of GO categories BP, CC, and MF in which the genes are involved.

The results of the GO analysis showing the biological process, cellular component, and molecular function in which the differentially expressed somatic mutated genes distinguishing the two patient subpopulations are summarized in Figure 7 for the most highly significant GO terms. GO analysis based on the biological processes revealed various biological processes (BP) in which the discovered genes are involved. For BP, the most significant GO categories included genes involved in nucleosome assembly and organization, immune response, DNA and protein complexes’ assembly and organization, and transport (Figure 7). Evaluation based on cellular component (CC) revealed genes involved in various cellular complexes, including the nucleosome, protein DNA, and potassium channel complexes. Evaluation based on molecular functions (MC) produced genes involved in various molecular function activities (Figure 7).

**Figure 8:**
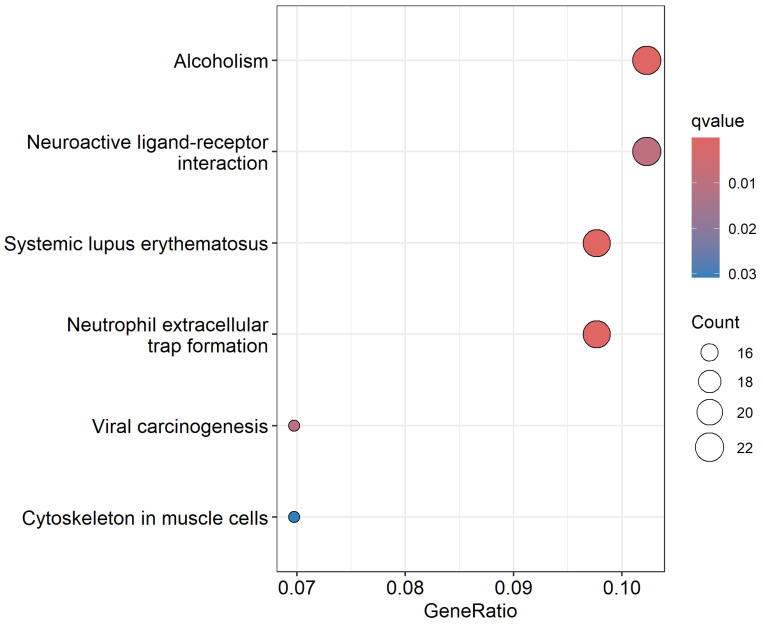
List of the most highly significant signaling pathways enriched for somatic mutations. This bubble plot highlights signaling pathways regulating significantly differentially expressed somatic mutated genes. The *x*-axis represents the gene ratio (proportion of genes involved in each pathway), while the color gradient indicates statistical significance based on q-values (adjusted *p*-values). The *y*-axis shows the names of signaling pathways enriched for somatic mutations. Larger bubbles reflect a higher gene count in each pathway.

The results of the pathway enrichment analysis are summarized in Figure 8, which presents a bubble plot of the most significantly enriched signaling pathways associated with somatic mutations in CRC. This analysis revealed several biologically important pathways, including those involved in alcoholism, neutrophil-mediated immune responses, viral carcinogenesis, and cytoskeleton regulation, all of which have established or emerging roles in colorectal cancer pathogenesis [62,63,64,65]. Notably, multiple genes from our integrative set of significantly differentially expressed and somatically mutated genes have been reported to be involved in these pathways [66,67,68,69]. For example, *TTN* and *ANKRD36*, both highly mutated in deceased patients and listed among the top-ranked genes (Table 2), are associated with cytoskeletal reorganization and structural maintenance, critical processes for tumor invasion and metastasis [69]. *CDH3* and *CLDN1*, which appear in the intersecting gene set, participate in cell adhesion and cytoskeletal signaling and have been widely reported as key mediators of CRC progression and metastatic potential [28,34]. Additionally, *ESM1* and *ETV4* are enriched in inflammatory and viral carcinogenesis pathways and play significant roles in remodeling the tumor microenvironment [31,70]. *ESM1* promotes angiogenesis and tumor progression in CRC through activation of the PI3K/Akt/mTOR signaling pathway and upregulation of pro-angiogenic and inflammatory factors such as VEGF, COX-2, and HIF-1α [31]. *ETV4*, in turn, enhances CRC cell invasion and metastasis by regulating matrix metalloproteinases and driving epithelial-to-mesenchymal transition via the ERK/EGFR signaling axis [70]. These findings collectively validate the biological relevance of our prioritized gene set and provide mechanistic insights into how somatic mutations and transcriptional dysregulation converge on key signaling pathways that drive CRC aggressiveness. The complete list of enriched pathways, gene ratios, and adjusted *p*-values is presented in Figure 8.

In this study, we leveraged gene expression data and integrated it with somatic mutation information for the discovery of potential diagnostic and prognostic biomarkers and potential therapeutic targets. The study demonstrates that CRC characterization efforts combining transcriptomic and mutational data could enhance colorectal cancer (CRC) characterization, offering unique opportunities to uncover clinically relevant biomarkers with diagnostic, prognostic, and therapeutic potential. By combining gene expression with somatic mutation information data generated on the same individuals, this study provides biological insights about CRC that could not be provided using gene expression and mutation datasets individually.

## 4. Discussion

The recent surge of next-generation sequencing of cancer genomes has enabled a rapid increase in omics datasets, creating unprecedented opportunities for integrating these genomic datasets to study the biological mechanisms underlying CRC biology and to discover potential biomarkers and therapeutic targets. In this study, we leveraged gene expression data and integrated it with somatic mutation information for the discovery of potential biomarkers and therapeutic targets in CRC. Our results demonstrate that integrating gene expression with somatic mutation data is an effective strategy for identifying potential diagnostic, prognostic, and therapeutic targets in CRC.

While several studies have explored the relationship between somatic mutations and gene expression in cancers such as breast, lung, and glioblastoma, including CRC, these efforts have largely focused on pan-cancer analyses or on individual driver mutations without the systematic integration of genome-wide mutation and expression profiles [71,72,73]. For example, integrative studies in breast cancer have shown that PIK3CA mutations correlate with specific transcriptional programs [74], and similar mutation–expression relationships have been identified for EGFR and KRAS in lung adenocarcinoma [73] and for IDH1 and TP53 in glioblastoma [72,73,74]. However, few studies have applied this integrative strategy in a comprehensive manner to colorectal cancer (CRC) using matched patient-level data. Our study advances this field by integrating transcriptomic and somatic mutation profiles from the same individuals to uncover dual-layered biomarkers associated with both tumor presence and patient survival. This approach allowed us to identify molecular networks and signaling pathways enriched for somatic mutations with potential diagnostic and prognostic value in CRC, an aspect not previously reported in this context.

A number of studies have reported the analysis of gene expression in CRC using RNA-Seq [75,76,77], including survival prediction [78,79] and somatic mutation analysis [80,81]. In addition, several studies have reported an integrative analysis of genomics data in colon cancer [6]. Muzny et al. reported a comprehensive molecular characterization of human colon and rectal cancer [6]. Kamran et al. showed integrative molecular characterization of resistance to neoadjuvant chemoradiation in rectal cancer [82]. The main difference between our study and these earlier reports is that our integrative approach addressed both the diagnostic stage by comparing tumor to control samples and the prognostic stage by comparing the dead versus alive using gene expression and somatic mutations generated on the same individuals. This has not been reported in previous reports. We did not investigate resistance to chemotherapy because such information was not available in our TCGA data used in our investigation. Overall, the novel aspect of our investigation is that it leverages gene expression data as an intermediate phenotype and integrates it with somatic mutations, providing novel insights that could not be provided by individual datasets.

Our findings revealed many somatic mutated candidate biomarkers and driver genes transcriptionally associated with CRC, including *CDH3*, *KRT80*, *ETV4*, *ESM1*, *FOXQ1*, *WNT2*, *CLDN1*, *AJUBA*, *NFE2L3*, *BEST4*, *CPNE7*, *INHBA*, *MTHFD1L*, *MMP7*, *PEX26*, *KLK6*, *TRIB3*, *KRT23*, *CEMIP*, *TRIP13*, *PHLPP2*, *SLCO4A1*, *MDFI*, *NOTUM*, *ENC1*, *VWA2*, and *LARGE2*, from tumors versus controls, which have been experimentally confirmed to be directly involved in CRC [28,29,30,31,32,33,34,35,36,37,38,39,40,41,42,43,44,45,46,47,48,49,50,51,52,53,54]. The potential translational impact of these findings is that these genes could serve as diagnostic biomarkers. For example, the *CDH3* has been shown to be overexpressed in CRC tumors and is reported to have the potential to serve as a serum biomarker for CRC monitoring [28]. *ETV4* plays a role in CRC progression [30], potentiating it as a prognostic biomarker. *ESM1* promotes angiogenesis in colorectal cancer by activating the PI3K/Akt/mTOR pathway, a mechanism that accelerates tumor progression [31]. Overexpression of *FOXQ1* in CRC has been shown to enhance tumor growth [32]. *CLDN1* has been reported as a novel biomarker for CRC [34]. Taken together, this evidence from the literature reports suggests that the discovered genes have the promise to serve as diagnostic and prognostic biomarkers.

Our findings in dead versus alive comparisons identified APC, TTN, and CDH3 as potential prognostic biomarkers in CRC [28,60,83]. The *APC* gene is a well-established tumor suppressor in CRC, playing a central role in regulating the Wnt signaling pathway and maintaining cellular homeostasis [33]. Mutations in *APC* are strongly associated with tumor initiation and progression in CRC. *TTN*, although historically known for its structural role in muscle tissue, has recently emerged as a biomarker of high tumor mutational burden (TMB) [28]. Studies have demonstrated that *TTN* mutations are frequently observed in CRC and other solid tumors and may reflect underlying genomic instability. High TMB has been associated with improved response to immunotherapy and worse baseline prognosis, especially in microsatellite-stable CRC cases [77]. Therefore, the *TTN* mutation status may serve as a surrogate prognostic indicator and inform treatment stratification. *CDH3* (P-cadherin), a calcium-dependent adhesion molecule, is frequently overexpressed in colorectal tumors [28]. Its role in promoting epithelial–mesenchymal transition (EMT) and enhancing tumor invasiveness has been well documented. *CDH3* overexpression has also been evaluated as a potential serum biomarker, making it a strong candidate for non-invasive prognostic monitoring in CRC patients [84,85]. Taken together, the somatic mutations in these genes and their functional involvement in key oncogenic processes highlight their translational potential as prognostic biomarkers and possible therapeutic targets in CRC.

The discovery of a signature of significantly differentially expressed genes distinguishing tumor from normal samples was consistent with published reports [86,87,88,89]. The innovative and novel aspect of our investigation is that the gene signatures and signaling pathways discovered in this study were enriched for somatic mutations and have not been previously reported. Of particular interest was the discovery of significantly differentially expressed somatic mutated genes distinguishing dead from alive samples. The discovered gene signature, if confirmed, could serve as a prognostic signature and a potential predictor of the clinical outcome.

Although, we did not perform a survival analysis due to limited data, evidence from published studies investigating the association between tumor gene expression patterns and patient survival outcomes has revealed several promising gene signatures that have demonstrated the ability to predict disease-free survival and overall survival in CRC patients, independent of the standard clinicopathological risk factors [84,85]. The novel aspect of our discovered prognostic gene signature is that it includes somatic driver mutations. As shown in the functional analysis in this report, these genes are crucial in fundamental biological processes, including cell cycle control, epithelial–mesenchymal transition, and immune regulation. The implementation of prognostic gene expression tests using somatic mutated genes in clinical practice holds great potential for enabling more personalized clinical management strategies for CRC. Importantly, the discovered genes could be used in the development of novel risk prediction algorithms, such as machine learning, to identify individuals at high risk of developing aggressive CRC, who could be prioritized for treatment. Although we did not develop and apply ML algorithms in this study due to limited data, others have reported the use of ML and gene expression data for predicting the survival of colorectal cancer patients [90,91,92]. However, it is worth noting that these studies did not involve the integration of gene expression data with somatic mutation information.

Our integrative analysis also uncovered several signaling pathways significantly enriched for somatic mutations, many of which are known to play roles in CRC pathogenesis [63,64,65,66]. These findings underscore the value of combining gene expression data with mutational profiling to identify potential novel therapeutic targets. Notably, pathways related to DNA damage response mechanisms, including those involving *CHEK1* and *CHEK2*, were among those enriched. These genes are key components of the DNA repair system and are targets of immune checkpoint inhibitors currently being developed and utilized in immunotherapy strategies for various malignancies, including CRC [93,94]. Taken together, the results show that integrative analysis combining gene expression with somatic mutation information is a powerful approach for the discovery of potential diagnostic and prognostic biomarkers and therapeutic targets in CRC.

Limitations: This study produced valuable insights about the benefits of integrating gene expression with somatic mutations for the discovery of potential clinically actionable diagnostic and prognostic biomarkers and potential therapeutic targets. However, the limitations must be acknowledged. Among the key limitations of our study are the lack of diversity in the study cohort, the lack of information on environmental perturbations, and the unbalanced study design with limited sample size. The majority of the samples used in this study were from individuals of European ancestry. However, it is generally well recognized from epidemiology studies that the incidence and mortality rates of CRC vary by race, ethnicity, and gender [1]. Additionally, epidemiology studies have shown that the development and progression of CRC arise from interactions between genes and the environment [1].

While addressing these factors was beyond the scope of this study, future investigations should aim to validate the findings in independent, multi-ethnic cohorts drawn from external sources such as the International Cancer Genome Consortium (ICGC), Gene Expression Omnibus (GEO), or large-scale biobanks that include underrepresented populations. Such efforts will enhance the reproducibility, external validation, and generalization of the identified biomarkers and ensure their translational relevance across diverse healthcare systems and patient populations. Additionally, we acknowledge the imbalance in sample sizes between the tumors and control samples and between the dead (n = 127) and alive (n = 495) tumor samples used in our integrative analyses. The unbalanced study design and limited sample size were due to the lack of additional accessible publicly available data with gene expression and somatic mutations on the same individuals and matched controls. While this limited our ability to apply propensity score matching or bootstrapping techniques, we addressed potential bias and potential false positives by applying DESeq2′s generalized linear model (GLM) with variance-stabilizing transformation to account for dispersion and covariate effects.

Indeed, many software packages have been developed for the identification of differentially expressed genes (DEGs) between treatment groups based on RNA-Seq data, and there is a lack of consensus on how to approach an optimal study design and choice of suitable software for the analysis. In this study, we used DESeq, because it has features that address unbalanced designs and biological replicates or sample size per group in RNA-Seq data analysis and has been shown to be superior to other tools [95]. Zhang et al. performed a comparative study that evaluated the performance of three of the most frequently used software tools for the analysis of RNA-Seq data, namely Cufflinks-Cuffdiff2, DESeq, and edgeR [95]. The study took into account a number of important parameters of RNA-Seq technology considerations, including the number of replicates or sample size, sequencing depth, and balanced versus unbalanced sequencing depth within and between groups. They also benchmarked the results relative to sets of DEGs identified through either quantitative RT-PCR or microarray. They concluded that, overall, the results from DESeq were recommended if the number of false positives is a major concern in the study. Our study used DESeq and the features implemented therein to address the problem of unbalanced study design and analysis. However, in the absence of complete consensus on the best tool or approach to completely address those problems, we acknowledge that future studies should leverage larger, more balanced, and clinically matched datasets to reduce potential confounding and to further validate the discovered biomarkers, depending on the availability of gene expression and somatic mutation data on the same individuals.

## 5. Conclusions

Integrative analysis combining gene expression with somatic mutation data produced a signature of significantly differentially expressed somatic mutated genes transcriptionally associated with CRC and a signature of significantly differentially expressed somatic mutated genes distinguishing dead from alive individuals. Functional analysis revealed molecular networks and signaling pathways enriched for somatic driver mutations. The study demonstrates that leveraging gene expression data and integrating it with somatic mutation data provides a powerful approach for the discovery of potential diagnostic and prognostic biomarkers and therapeutic targets. Further research integrating multiomics data with epigenetics data from diverse populations is recommended to understand the full spectrum of CRC pathogenesis in diverse populations.

## Figures and Tables

**Table 1:** List of the top 40 most highly significantly differentially expressed somatically mutated genes (tumor versus control), along with their chromosomal positions, estimates of *p*-values, log2 fold changes, and the number of somatic mutations observed per gene, the references showing evidence of the genes’ involvement in CRC.

Gene Symbol	Chromosome Position	Log2FC	*p*-Value	Correlation	Total Mutations	Involvement in CRC [REF]
***CDH3***	16q22.1	5.66	1.00 × 10^−300^	0.95	16	[28]
***KRT80***	12q13.13	6.56	1.00 × 10^−300^	0.93	9	[29]
***ETV4***	17q21.31	5.14	3.57 × 10^−257^	0.93	18	[30]
***ESM1***	5q11.2	5.71	3.57 × 10^−257^	0.92	10	[31]
***FOXQ1***	6p25.3	6.32	6.37 × 10^−238^	0.90	11	[32]
*SIM2*	21q22.13	7.40	4.62 × 10^−225^	0.88	15	
***WNT2***	7q31.2	5.63	5.59 × 10^−225^	0.90	16	[33]
***CLDN1***	3q28	4.72	1.54 × 10^−222^	0.92	2	[34]
***AJUBA***	14q11.2	2.96	5.42 × 10^−199^	0.92	9	[35]
***NFE2L3***	7p15.2	2.73	2.50 × 10^−175^	0.91	26	[36]
***BEST4***	1p34.1	−5.84	1.72 × 10^−168^	−0.94	12	[37]
***CPNE7***	16q24.3	5.73	5.78 × 10^−166^	0.87	20	[38]
***INHBA***	7p14.1	5.43	6.09 × 10^−160^	0.89	46	[39]
*CST1*	20p11.21	8.2	9.70 × 10^−157^	0.82	19	
*EPHX4*	1p22.1	4.42	1.30 × 10^−156^	0.87	18	
*APPL2*	12q23.3	−1.86	8.28 × 10^−155^	−0.86	18	
***MTHFD1L***	6q25.1	2.15	1.23 × 10^−154^	0.90	37	[40]
***MMP7***	11q22.2	7.09	1.95 × 10^−147^	0.85	15	[41]
***PEX26***	22q11.21	−1.74	2.86 × 10^−147^	−0.80	5	[42]
*C6orf223*	6p21.	14.50	7.15 × 10^−147^	0.89	5	
*SLC51A*	3q29	−3.90	1.16 × 10^−141^	−0.74	12	
*GRIN2D*	19q13.33	4.81	1.04 × 10^−138^	0.84	38	
***KLK6***	19q13.41	9.70	5.94 × 10^−136^	0.77	16	[43]
*LRP8*	1p32.3	2.89	7.07 × 10^−134^	0.89	23	
*TRIB3*	20p13	3.75	5.24 × 10^−132^	0.87	13	[44]
*GLTP*	12q24.11	−1.63	1.06 × 10^−130^	−0.87	4	
*KRT23*	17q21.2	7.32	2.84 × 10^−128^	0.76	15	[45]
*COL11A1*	1p21.1	5.99	1.52 × 10^−127^	0.84	106	
***CEMIP***	15q25.1	4.12	2.22 × 10^−127^	0.88	41	[46]
***TRIP13***	5p15.33	2.15	1.35 × 10^−121^	0.88	7	[47]
***PHLPP2***	16q22.2	−2.58	2.89 × 10^−121^	−0.85	30	[48]
*SLC25A34*	1p36.21	−3.79	5.15 × 10^−120^	−0.88	11	
***SLCO4A1***	20q13.33	3.41	6.26 × 10^−120^	0.87	23	[49]
***MDFI***	6p21.1	3.58	6.26 × 10^−120^	0.86	4	[50]
***NOTUM***	17q25.3	8.67	1.72 × 10^−119^	0.73	18	[51]
***ENC1***	5q13.3	2.01	3.55 × 10^−119^	0.88	28	[52]
***VWA2***	10q25.3	3.94	1.25 × 10^−118^	0.84	26	[53]
*SLC22A5*	5q31.1	−1.93	2.19 × 10^−117^	−0.82	8	
***LARGE2***	11p11.2	4.37	3.04 × 10^−117^	0.84	17	[54]
*ETFDH*	4q32.1	−1.84	7.55 × 10^−116^	−0.90	13	

Negative log2FC and correlation indicate downregulation, and positive indicates upregulation. Genes in bold fonts have been experimentally confirmed to be involved in CRC. RF indicates the reference providing evidence of the gene’s direct involvement in the CRC cited.

**Table 2:** Top 40 most highly significantly differentially expressed genes enriched for somatic mutations distinguishing tumor samples that were alive and tumor samples that were deceased.

Gene	Chromosome Position	Adjusted *p*-Value	Log2FC	Correlation	Mutations in Alive (n = 495)	Mutations in Deceased (n = 127) [RF]
*H3C2*	6p22.2	2.19 × 10^−49^	−4.68	−0.20	6	3
*H2BC13*	6p22.1	1.86 × 10^−47^	−3.88	−0.24	6	1
*H1-3*	6p22.2	6.03 × 10^−46^	−4.44	−0.18	7	3
*H2BC17*	6p22.1	7.36 × 10^−41^	−3.99	−0.21	1	0
*H2AC13*	6p22.1	2.03 × 10^−38^	−3.42	−0.23	3	0
*H1-4*	6p22.2	2.31 × 10^−38^	−3.97	−0.19	7	1
*ZBTB20*	3q13.31	4.66 × 10^−36^	−2.82	−0.16	39	15
*ZNF460*	19q13.43	6.90 × 10^−34^	−2.58	−0.20	11	6
*H4C5*	6p22.2	2.43 × 10^−30^	−2.98	−0.16	2	2
*LRRTM2*	5q31.2	2.92 × 10^−30^	−2.82	−0.17	14	1
*H1-5*	6p22.1	4.73 × 10^−30^	−3.57	−0.19	15	0
*OMG*	17q11.2	3.82 × 10^−28^	−2.80	−0.16	4	0
*ANKRD36C*	2q11.1	2.70 × 10^−24^	−2.17	−0.14	3	0
*H2AC20*	1q21.2	5.25 × 10^−24^	−2.30	−0.20	7	2
*H2BC7*	6p22.2	4.08 × 10^−22^	−2.53	−0.15	2	4
*H2BC18*	1q21.2	7.01 × 10^−21^	−2.32	−0.11	5	1
*GSN-AS1*	9q33.2	3.24 × 10^−20^	−2.09	−0.16	5	2
*NBEAL1*	2q33.2	3.76 × 10^−20^	−1.59	−0.15	29	9
*GPR82*	Xp11.4	−1.19 × 10^−19^	−1.73	−0.23	5	0
*ANKRD36B*	2q11.2	1.18 × 10^−18^	−1.65	−0.16	10	1
***TTN***	2q31.2	1.90 × 10^−18^	−1.86	−0.12	360	95 [62]
*GRIK1*	21q21.3	4.26 × 10^−18^	−1.99	−0.16	35	4
*GPR18*	13q32.3	6.94 × 10^−18^	−1.82	−0.16	6	2
*SYCP3*	12q23.2	7.08 × 10^−18^	−1.83	−0.13	5	0
*CLDN20*	6q25.3	1.10 × 10^−17^	−1.97	−0.15	3	1
*INSYN2A*	10q26.2	1.12 × 10^−17^	−2.03	−0.14	8	6
***CCDC144B***	17p11.2	3.37 × 10^−17^	−2.57	−0.13	4	1 [63]
*GNAT2*	1p13.3	4.38 × 10^−17^	−1.42	−0.25	10	3
*H2BC4*	6p22.2	6.24 × 10^−17^	−1.88	−0.16	8	2
*GCNT7*	20q13.31	1.53 × 10^−16^	−1.88	−0.14	3	1
*H2BC6*	6p22.2	2.50 × 10^−16^	−1.86	−0.15	3	3
*KCNC1*	10q25.3	3.75 × 10^−16^	−1.91	−0.11	19	8
***TDRD1***	10q25.3	4.57 × 10^−16^	−2.15	−0.10	16	5 [64]
*SPDYE1*	7p13	4.61 × 10^−16^	−1.74	−0.12	7	2
***ANKRD36***	2q11.2	2.29 × 10^−15^	−1.49	−0.13	4	2 [65]
*LRRN3*	7q31.1	2.66 × 10^−15^	−1.81	−0.15	32	7
*MAK*	6p24.2	2.94 × 10^−15^	−1.57	−0.13	15	6
*H2AC11*	6p22.1	4.82 × 10^−15^	−1.53	−0.17	8	2
*SHOC1*	9q31.3	9.09 × 10^−15^	−2.30	−0.14	20	9
*COL6A6*	3q22.1	1.76 × 10^−14^	−1.90	−0.13	67	11

Genes in bold have been previously associated with CRC. Negative log2FC indicates downregulation, and positive indicates upregulation. Gene in bold fonts have been experimentally confirmed to be involved in CRC, and RF indicates references cited.

## Data Availability

Original RNA-Seq, somatic mutation, and clinical data used in this investigation are available and downloadable from the Genomics Data Commons: https://portal.gdc.cancer.gov/. All the data results from this study are included in the Supplementary Tables and are downloadable from the “MDPI Research Data Policies” at https://www.mdpi.com/ethics.

## Supplementary Materials

The following supporting information can be downloaded at https://www.mdpi.com/article/10.3390/biomedicines13071651/s1: Figure S1: Volcano plot of significantly differentially expressed somatic mutated genes resulting from comparing gene expression values between tumor and control samples. Points colored red are significantly upregulated, points colored blue are significantly downregulated, and points colored gray are not significantly differentially expressed. Figure S2: Volcano plot of significantly differentially expressed and somatic mutated genes resulting from comparing gene expression values between dead and alive samples. Points highlighted in red are significantly upregulated, points highlighted in blue are significantly downregulated, and points highlighted in gray are not significant. Table S1: List of all somatic mutated genes organized by tumor samples and sample ID in the dead and alive samples used in this study. SNP = single-nucleotide polymorphism, INS = insert, and DEL = deletion. Table S2: List of significantly differentially expressed and somatic mutated genes transcriptionally associated with CRC from the analysis comparing gene expression values between the tumor and control samples. Table S3: List of significantly differentially expressed and differentially somatic mutated genes transcriptionally associated with CRC from the analysis comparing gene expression values between the dead and alive samples.

## Abbreviations

The following abbreviations are used in this manuscript:

CRC	Colorectal cancer
AACR	American Association of Cancer Research (AACR)
GLOBOCAN	The Global Cancer Observatory
TCGA	Cancer Genome Atlas
ICGC	International Cancer Genome Consortium
RNA-Seq	RNA sequencing
GDC	Genomics Data Commons
SNP	Single-nucleotide polymorphisms
INS	Insert
DEL	Deletion
Indel	Insert and deletion
MAF	Mutation Annotation Format
N	Number of samples
VST	variance stabilizing transformation
GLM	Generalized linear model
DEGs	Differentially expressed genes
Log2FC	Log base 2 fold changes
FDR	False discover rate
GO	Gene Ontology
IPA	Ingenuity pathway analysis
REF	Reference cited
BP	Biological process
CC	Cellular component
MF	Molecular function

## References

[1] Siegel R.L., Giaquinto A.N., Jemal A. (2024). Cancer Statistics, 2024. CA Cancer J. Clin..

[2] Bray F., Laversanne M., Sung H., Ferlay J., Siegel R.L., Soerjomataram I., Jemal A. (2024). Global cancer statistics 2022: GLOBOCAN estimates of incidence and mortality worldwide for 36 cancers in 185 countries. CA Cancer J Clin..

[3] Morgan E., Arnold M., Gini A., Lorenzoni V., Cabasag C.J., Laversanne M., Vignat J., Ferlay J., Murphy N., Bray F. (2023). Global Burden of Colorectal Cancer in 2020 and 2040: Incidence and Mortality Estimates from GLOBOCAN. Gut.

[4] Hossain M.S., Karuniawati H., Jairoun A.A., Urbi Z., Ooi D.J., John A., Lim Y.C., Kibria K.M., Mohiuddin A.K.M., Ming L.C. (2022). Colorectal Cancer: A Review of Carcinogenesis, Global Epidemiology, Current Challenges, Risk Factors, Preventive and Treatment Strategies. Cancers.

[5] Dang Q., Chen Y., Bai X., Liu H., Zhang J., Fan Y., Luo A., Sun K., Li X. (2024). Molecular Subtypes of Colorectal Cancer in the Era of Precision Oncotherapy: Current Inspirations and Future Challenges. Cancer Med..

[6] The Cancer Genome Atlas Network (2012). Comprehensive Molecular Characterization of Human Colon and Rectal Cancer. Nature.

[7] Tomczak K., Czerwińska P., Wiznerowicz M. (2015). The Cancer Genome Atlas (TCGA): An Immeasurable Source of Knowledge. Contemp. Oncol..

[8] Lathrop M., Gut I., Heath S., Tost J., Gress T., Hudson T. (2010). International Network of Cancer Genome Projects. Nature.

[9] Ullah I., Yang L., Yin F.T., Sun Y., Li X.H., Li J., Wang X.J. (2022). Multi-Omics Approaches in Colorectal Cancer Screening and Diagnosis, Recent Updates and Future Perspectives. Cancers.

[10] Lee J.H., Ahn B.K., Baik S.S., Lee K.H. (2019). Comprehensive Analysis of Somatic Mutations in Colorectal Cancer with Peritoneal Metastasis. Vivo.

[11] Hassan S., Khatoon A., Bukhari U., Mirza T. (2023). Analysis of Common Somatic Mutations in Colorectal Carcinoma and Associated Dysregulated Pathways. J. Ayub Med. Coll. Abbottabad.

[12] Yamada S., Osakabe M., Uesugi N., Yanagawa N., Matsumoto T., Suzuki H., Sugai T. (2023). Genome-Wide Analysis of Colorectal Cancer Based on Gene-Based Somatic Copy Number Alterations during Neoplastic Progression within the Same Tumor. Cancer Med..

[13] Carvalho B., Diosdado B., Terhaar Sive Droste J.S., Bolijn A.S., Komor M.A., de Wit M., Bosch L.J.W., van Burink M., Dekker E., Kuipers E.J. (2018). Evaluation of Cancer-Associated DNA Copy Number Events in Colorectal (Advanced) Adenomas. Cancer Prev. Res..

[14] Weinstein J.N., Collisson E.A., Mills G.B., Shaw K.R.M., Ozenberger B.A., Ellrott K., Shmulevich I., Sander C., Stuart J.M. (2013). The Cancer Genome Atlas Pan-Cancer Analysis Project. Nat. Genet..

[15] National Cancer Institute (2024). Genomic Data Commons (GDC) Data Portal. https://gdc.cancer.gov/.

[16] Grossman R.L., Heath A.P., Ferretti V., Varmus H.E., Lowy D.R., Kibbe W.A., Staudt L.M. (2016). Toward a Shared Vision for Cancer Genomic Data. N. Engl. J. Med..

[17] Mayakonda A., Lin D.-C., Assenov Y., Plass C., Koeffler H.P. (2018). Maftools: Efficient and Comprehensive Analysis of Somatic Variants in Cancer. Genome Res..

[18] Mamidi T.K.K., Wu J., Hicks C. (2021). Elucidation of the Genomic-Epigenomic Interaction Landscape of Aggressive Prostate Cancer. BioMed Res. Int..

[19] Smedley D., Haider S., Durinck S., Pandini L., Provero P., Allen J., Arnaiz O., Awedh M.H., Baldock R., Barbiera G. (2015). The BioMart Community Portal: An Innovative Alternative to Large, Centralized Data Repositories. Nucleic Acids Res..

[20] Love M.I., Huber W., Anders S. (2014). Moderated Estimation of Fold Change and Dispersion for RNA-Seq Data with DESeq2. Genome Biol..

[21] Wald A. (1943). Tests of Statistical Hypotheses Concerning Several Parameters When the Number of Observations is Large. Trans. Am. Math. Soc..

[22] Benjamini Y., Hochberg Y. (1995). Controlling the False Discovery Rate: A Practical and Powerful Approach to Multiple Testing. J. R. Stat. Soc. Ser. B (Methodol.).

[23] Raivo Kolde Pheatmap: Pretty Heatmaps 2010, 1.0.12. https://cran.r-project.org/web/packages/pheatmap/index.html.

[24] Ashburner M., Ball C.A., Blake J.A., Botstein D., Butler H., Cherry J.M., Davis A.P., Dolinski K., Dwight S.S., Eppig J.T. (2000). Gene Ontology: Tool for the Unification of Biology. Nat. Genet..

[25] Yu G., Wang L.-G., Han Y., He Q.-Y. (2012). clusterProfiler: An R package for comparing biological themes among gene clusters. OMICS.

[26] Ge S.X., Jung D., Yao R. (2020). ShinyGO: A graphical gene-set enrichment tool for animals and plants. Bioinformatics.

[27] QIAGEN (2024). Ingenuity Pathways Analysis (IPA) System. http://www.ingenuity.com/.

[28] Kumara H.M.C.S., Bellini G.A., Caballero O.L., Herath S.A.C., Su T., Ahmed A., Njoh L., Cekic V., Whelan R.L. (2017). P-Cadherin (CDH3) is overexpressed in colorectal tumors and has potential as a serum marker for colorectal cancer monitoring. Oncoscience.

[29] Lin J., Fan X., Chen J., Xie X., Yu H. (2020). Small Interfering RNA-Mediated Knockdown of KRT80 Suppresses Colorectal Cancer Proliferation. Exp. Ther. Med..

[30] Fonseca A.S., Ramão A., Bürger M.C., De Souza J.E.S., Zanette D.L., De Molfetta G.A., De Araújo L.F., De Barros E Lima Bueno R., Aguiar G.M., Plaça J.R. (2021). ETV4 plays a role on the primary events during the adenoma-adenocarcinoma progression in colorectal cancer. BMC Cancer.

[31] Yang L., Dong Z., Li S., Chen T. (2023). ESM1 promotes angiogenesis in colorectal cancer by activating PI3K/Akt/mTOR pathway, thus accelerating tumor progression. Aging.

[32] Kaneda H., Arao T., Tanaka K., Tamura D., Aomatsu K., Kudo K., Sakai K., De Velasco M.A., Matsumoto K., Fujita Y. (2010). FOXQ1 Is Overexpressed in Colorectal Cancer and Enhances Tumorigenicity and Tumor Growth. Cancer Res..

[33] Jung Y.-S., Jun S., Lee S.-H., Sharma A., Park J.-I. (2015). Wnt2 Complements Wnt/Beta-Catenin Signaling in Colorectal Cancer. Oncotarget.

[34] Nakagawa S., Miyoshi N., Ishii H., Mimori K., Tanaka F., Sekimoto M., Doki Y., Mori M. (2011). Expression of CLDN1 in Colorectal Cancer: A Novel Marker for Prognosis. Int. J. Oncol..

[35] Wu Z., Zou X., Xu Y., Zhou F., Kuai R., Li J., Yang D., Chu Y., Peng H. (2021). AJUBA Transactivates N-Cadherin Expression in Colorectal Cancer Cells through Interaction with Twist. J. Cell. Mol. Med..

[36] Saliba J., Coutaud B., Makhani K., Epstein Roth N., Jackson J., Park J.-Y., Gagnon N., Costa P., Jeyakumar T., Bury M. (2022). Loss of NFE2L3 Protects against Inflammation-Induced Colorectal Cancer through Modulation of the Tumor Microenvironment. Oncogene.

[37] He X., Ye W., Zhang Y., Yang X., Liu F., Wang J., Ding X., Yang Y., Zhang R., Zhao Y. (2022). Oncogenic Potential of BEST4 in Colorectal Cancer via Activation of PI3K/Akt Signaling. Oncogene.

[38] Kong H.J., Kang D.H., Ahn T.S., Kim K.S., Kim T.W., Lee S.H., Lee D.W., Ryu J.S., Beak M.J. (2023). The Role of CPNE7 (Copine-7) in Colorectal Cancer Prognosis and Metastasis. Int. J. Mol. Sci..

[39] Okano M., Yamamoto H., Ohkuma H., Kano Y., Kim H., Nishikawa S., Konno M., Kawamoto K., Haraguchi N., Takemasa I. (2013). Significance of INHBA expression in human colorectal cancer. Oncol. Rep..

[40] Agarwal S., Behring M., Hale K., Al Diffalha S., Wang K., Manne U., Varambally S. (2019). MTHFD1L, A Folate Cycle Enzyme, Is Involved in Progression of Colorectal Cancer. Transl. Oncol..

[41] Zhou Y., Wang L., Zhou F. (2023). Clinical Significance of MMP7 Levels in Colorectal Cancer Patients Receiving FOLFOX4 Chemotherapy Treatment. Int. J. Gen. Med..

[42] Yan B., Cao L., Gao L., Wei S., Wang M., Tian Y., Yang J., Chen E. (2024). PEX26 Functions as a Metastasis Suppressor in Colorectal Cancer. Dig. Dis. Sci..

[43] Christodoulou S., Alexopoulou D.K., Kontos C.K., Scorilas A., Papadopoulos I.N. (2014). Kallikrein-Related Peptidase-6 (KLK6) mRNA Expression Is an Independent Prognostic Tissue Biomarker of Poor Disease-Free and Overall Survival in Colorectal Adenocarcinoma. Tumor Biol..

[44] Shang S., Yang Y.-W., Chen F., Yu L., Shen S.-H., Li K., Cui B., Lv X.-X., Zhang C., Yang C. (2022). TRIB3 Reduces CD8+ T Cell Infiltration and Induces Immune Evasion by Repressing the STAT1-CXCL10 Axis in Colorectal Cancer. Sci. Transl. Med..

[45] Birkenkamp-Demtröder K., Hahn S.A., Mansilla F., Thorsen K., Maghnouj A., Christensen R., Øster B., Ørntoft T.F. (2013). Keratin23 (KRT23) Knockdown Decreases Proliferation and Affects the DNA Damage Response of Colon Cancer Cells. PLoS ONE.

[46] Domanegg K., Sleeman J.P., Schmaus A. (2022). CEMIP, a Promising Biomarker That Promotes the Progression and Metastasis of Colorectal and Other Types of Cancer. Cancers.

[47] Agarwal S., Behring M., Kim H.G., Chandrashekar D.S., Chakravarthi B.V.S.K., Gupta N., Bajpai P., Elkholy A., Al Diffalha S., Datta P.K. (2020). TRIP13 Promotes Metastasis of Colorectal Cancer Regardless of P53 and Microsatellite Instability Status. Mol. Oncol..

[48] Wu S., Xu X., Sun C., Wen F., He S., Gao X., Liu Y., Liu L. (2019). Expression of PHLPP2 Correlates with Clinicopathologic Characteristics and Prognosis in Colorectal Cancer. Int. J. Clin. Exp. Pathol..

[49] Chen X., Yi G., Zhou Y., Hu W., Xi L., Han W., Wang F. (2023). Prognostic Biomarker SLCO4A1 Is Correlated with Tumor Immune Infiltration in Colon Adenocarcinoma. Mediat. Inflamm..

[50] Ma D., Liu S., Liu K., Kong L., Xiao L., Xin Q., Jiang C., Wu J. (2024). MDFI Promotes the Proliferation and Tolerance to Chemotherapy of Colorectal Cancer Cells by Binding ITGB4/LAMB3 to Activate the AKT Signaling Pathway. Cancer Biol. Ther..

[51] Yoon J.-H., Kim D., Kim J., Lee H., Ghim J., Kang B.-J., Song P., Suh P.-G., Ryu S.H., Lee T.G. (2018). NOTUM Is Involved in the Progression of Colorectal Cancer. Cancer Genom. Proteom..

[52] Cui Y., Yang J., Bai Y., Li Q., Yao Y., Liu C., Wu F., Zhang J., Zhang Y. (2021). ENC1 Facilitates Colorectal Carcinoma Tumorigenesis and Metastasis via JAK2/STAT5/AKT Axis-Mediated Epithelial Mesenchymal Transition and Stemness. Front. Cell Dev. Biol..

[53] Tian Y., Zhao Q., Wu H., Guo J., Wu H. (2024). VWA2 Protein Molecular Mechanism Predicts Colorectal Cancer: Promoting Cell Invasion and Migration by Inhibiting NK Cell Activation. Int. J. Biol. Macromol..

[54] Dietinger V., García de Durango C.R., Wiechmann S., Boos S.L., Michl M., Neumann J., Hermeking H., Kuster B., Jung P. (2020). Wnt-Driven LARGE2 Mediates Laminin-Adhesive O-Glycosylation in Human Colonic Epithelial Cells and Colorectal Cancer. Cell Commun. Signal..

[55] Zhao A., Zhu X., Wu H., Wang J., Zhang M., Xiang J., Xia S., Shi T., Xi Q. (2024). B7-H3 Promotes the Migration and Invasion of Colorectal Cancer Cells via Regulating the Actin Cytoskeleton and RhoA/ROCK1/LIMK1 Signaling Pathway. Tissue Cell.

[56] Caliceti C., Punzo A., Silla A., Simoni P., Roda G., Hrelia S. (2022). New Insights into Bile Acids Related Signaling Pathways in the Onset of Colorectal Cancer. Nutrients.

[57] Yu C., Chen F., Jiang J., Zhang H., Zhou M. (2019). Screening Key Genes and Signaling Pathways in Colorectal Cancer by Integrated Bioinformatics Analysis. Mol. Med. Rep..

[58] Zuo L., Yang X., Lu M., Hu R., Zhu H., Zhang S., Zhou Q., Chen F., Gui S., Wang Y. (2016). All-Trans Retinoic Acid Inhibits Human Colorectal Cancer Cells RKO Migration via Downregulating Myosin Light Chain Kinase Expression through MAPK Signaling Pathway. Nutr. Cancer.

[59] Cao L., Ba Y., Chen F., Li D., Zhang S., Zhang H. (2025). The prognostic significance of epoxide hydrolases in colorectal cancer. Biochem. Biophys. Rep..

[60] Drews R.M., Hernando B., Tarabichi M., Haase K., Lesluyes T., Smith P.S., Morrill Gavarró L., Couturier D.L., Liu L., Schneider M. (2022). A pan-cancer compendium of chromosomal instability. Nature.

[61] Zhao L., Fan W., Luo K., Xie S., Wang R., Guan J., Chen Z., Jin S. (2023). Construction of a TTN Mutation-Based Prognostic Model for Evaluating Immune Microenvironment, Cancer Stemness, and Outcomes of Colorectal Cancer Patients. Stem Cells Int..

[62] Kim S.T., Sohn I., DO I.-G., Jang J., Kim S.H., Jung I.H., Park J.O., Park Y.S., Talasaz A., Lee J. (2014). Transcriptome Analysis of CD133-Positive Stem Cells and Prognostic Value of CCDC144B Survivin in Colorectal Cancer. Cancer Genom. Proteom..

[63] Ha Y.M., Choi E.J., Yoo N.J., Lee S.H. (2020). Mutational Alterations of TDRD1, 4 and 9 Genes in Colorectal Cancers. Pathol. Oncol. Res..

[64] Huang L., Zou T., Liang W., Mo C., Wei J., Deng Y., Ou M. (2023). High-Throughput Sequencing Reveals That Rotundine Inhibits Colorectal Cancer by Regulating Prognosis-Related Genes. J. Pers. Med..

[65] Qiu T., Hu W., Rao Z., Fang T. (2022). The Molecular Basis of the Associations Between Non-Alcoholic Fatty Liver Disease and Colorectal Cancer. Front. Genet..

[66] Li C., Chen T., Liu J., Wang Y., Zhang C., Guo L., Shi D., Zhang T., Wang X., Li J. (2023). FGF19-Induced Inflammatory CAF Promoted Neutrophil Extracellular Trap Formation in the Liver Metastasis of Colorectal Cancer. Adv. Sci..

[67] Ivancevic A., Simpson D.M., Joyner O.M., Bagby S.M., Nguyen L.L., Bitler B.G., Pitts T.M., Chuong E.B. (2024). Endogenous Retroviruses Mediate Transcriptional Rewiring in Response to Oncogenic Signaling in Colorectal Cancer. Sci. Adv..

[68] Anupriya S., Parida N., Patnaik S. (2024). SOX4 Induces Cytoskeleton Remodeling and Promotes Cell Motility via N-Wasp/ARP2/3 Pathway in Colorectal Cancer Cells. Exp. Cell Res..

[69] Wei H., Ren K., Zhang Q., Jin Y., Cao B., Tian Z., Mao T., Ren L. (2023). Titin as a potential novel therapeutic target in colorectal cancer. J. Cell. Mol. Med..

[70] Leng Y., Chen Z., Ding H., Zhao X., Qin L., Pan Y. (2021). Overexpression of microRNA-29b inhibits epithelial-mesenchymal transition and angiogenesis of colorectal cancer through the ETV4/ERK/EGFR axis. Cancer Cell Int..

[71] Kim H., Kim Y.-M. (2018). Pan-cancer analysis of somatic mutations and transcriptomes reveals common functional gene clusters shared by multiple cancer types. Sci. Rep..

[72] Verhaak R.G.W., Hoadley K.A., Purdom E., Wang V., Qi Y., Wilkerson M.D., Miller C.R., Ding L., Golub T., Mesirov J.P. (2010). Integrated Genomic Analysis Identifies Clinically Relevant Subtypes of Glioblastoma Characterized by Abnormalities in PDGFRA, IDH1, EGFR, and NF1. Cancer Cell.

[73] The Cancer Genome Atlas Research Network (2014). Comprehensive molecular profiling of lung adenocarcinoma. Nature.

[74] Zardavas D., Phillips W.A., Loi S. (2014). PIK3CA mutations in breast cancer: Reconciling findings from preclinical and clinical data. Breast Cancer Res..

[75] Slattery M.L., Pellatt A.J., Mullany L.E., Wolff R.K., Herrick J.S. (2015). Gene Expression in Colon Cancer: A Focus on Tumor Site and Molecular Phenotype. Genes Chromosomes Cancer.

[76] Zuo Z., Zhang X., Ye X., Zhou Z., Wu X., Ni S., Song H. (2016). Bioinformatic Analysis of RNA-Seq Data Unveiled Critical Genes in Rectal Adenocarcinoma. Eur. Rev. Med. Pharmacol. Sci..

[77] Elsayed A.M.A., Oweda M., Abushady A.M., Alhelf M., Khalil S.R.M., Tawfik M.S., Al-Atabany W., El-Hadidi M. (2024). Identification of Differentially Expressed Genes in Human Colorectal Cancer Using RNASeq Data Validated on the Molecular Level with Real-Time PCR. Biochem. Genet..

[78] Liu R., Zhang W., Liu Z.-Q., Zhou H.-H. (2017). Associating Transcriptional Modules with Colon Cancer Survival through Weighted Gene Co-Expression Network Analysis. BMC Genom..

[79] Li H., Peng J., Wang Y., Jiang H., Wang J. (2022). A Novel Nomogram Associated with Regulatory T Cells Infiltration by Weighted Gene Co-Expression Network Analysis for Predicting Survival in Patients with Colon Cancer. Eur. Rev. Med. Pharmacol. Sci..

[80] Almuzzaini B., Alghamdi J., Alomani A., AlGhamdi S., Alsharm A.A., Alshieban S., Sayed A., Alhejaily A.G., Aljaser F.S., Abudawood M. (2021). Identification of Novel Mutations in Colorectal Cancer Patients Using AmpliSeq Comprehensive Cancer Panel. J. Pers. Med..

[81] Chen J., Apizi A., Wang L., Wu G., Zhu Z., Yao H., Chen G., Shi X., Shi B., Tai Q. (2021). TCGA Database Analysis of the Tumor Mutation Burden and Its Clinical Significance in Colon Cancer. J. Gastrointest. Oncol..

[82] Kamran S.C., Lennerz J.K., Margolis C.A., Liu D., Reardon B., Wankowicz S.A., Van Seventer E.E., Tracy A., Wo J.Y., Carter S.L. (2019). Integrative Molecular Characterization of Resistance to Neoadjuvant Chemoradiation in Rectal Cancer. Clin. Cancer Res..

[83] Hankey W., Frankel W.L., Groden J. (2018). Functions of the APC tumor suppressor protein dependent and independent of canonical WNT signaling: Implications for therapeutic targeting. Cancer Metastasis Rev..

[84] Saadh M.J., Allela O.Q.B., Kareem R.A., Baldaniya L., Ballal S., Vashishth R., Parmar M., Sameer H.N., Hamad A.K., Athab Z.H. (2025). Prognostic Gene Expression Profile of Colorectal Cancer. Gene.

[85] Abdul Aziz N.A., Mokhtar N.M., Harun R., Mollah M.M., Rose I.M., Sagap I., Tamil A.M., Ngah W.Z., Jamal R. (2016). A 19-Gene Expression Signature as a Predictor of Survival in Colorectal Cancer. BMC Med. Genom..

[86] Wei I.H., Shi Y., Jiang H., Kumar-Sinha C., Chinnaiyan A.M. (2014). RNA-Seq accurately identifies cancer biomarker signatures to distinguish tissue of origin. Neoplasia.

[87] Johnson E., Theisen C.S., Johnson K.R., Wheelock M.J. (2004). R-cadherin Influences Cell Motility via Rho Family GTPases. J. Biol. Chem..

[88] Gonzalez-Pons M., Cruz-Correa M. (2015). Colorectal Cancer Biomarkers: Where Are We Now?. BioMed Res. Int..

[89] Kalmár A., Wichmann B., Galamb O., Spisák S., Tóth K., Leiszter K., Nielsen B.S., Barták B.K., Tulassay Z., Molnár B. (2015). Gene-Expression Analysis of a Colorectal Cancer-Specific Discriminatory Transcript Set on Formalin-Fixed, Paraffin-Embedded (FFPE) Tissue Samples. Diagn. Pathol..

[90] Khaneh A., Piri H., Alaei M., Parvani N., Vakilzadeh I., Javadi S., Moradian Haft Cheshmeh Z., Razzaghi Z., Robati R.M., Zamanian Azodi M. (2024). Classification and Diagnostic Prediction of Colorectal Cancer Mortality Based on Machine Learning Algorithms: A Multicenter National Study. Asian Pac. J. Cancer Prev..

[91] Maurya N.S., Kushwaha S., Vetukuri R.R., Mani A. (2023). Unlocking the Potential of the CA2, CA7, and ITM2C Gene Signatures for the Early Detection of Colorectal Cancer: A Comprehensive Analysis of RNA-Seq Data by Utilizing Machine Learning Algorithms. Genes.

[92] Li J., Wang P., Zhou Y., Liang H., Luan K. (2021). Different Machine Learning and Deep Learning Methods for the Classification of Colorectal Cancer Lymph Node Metastasis Images. Front. Bioeng. Biotechnol..

[93] Kassab R., Khalil M.A., Kassab J., Kourie H.R. (2023). Immune Checkpoint Inhibitors in BRAF-Mutated Advanced Colorectal Cancer. Future Oncol..

[94] Ghidini M., Fusco N., Salati M., Khakoo S., Tomasello G., Petrelli F., Trapani D., Petrillo A. (2021). The Emergence of Immune-Checkpoint Inhibitors in Colorectal Cancer Therapy. Curr. Drug Targets.

[95] Zhang Z.H., Jhaveri D.J., Marshall V.M., Bauer D.C., Edson J., Narayanan R.K., Robinson G.J., Lundberg A.E., Bartlett P.F., Wray N.R. (2014). A comparative study of techniques for differential expression analysis on RNA-Seq data. PLoS ONE.

