# Supplementary material for: An Integrative Genomics Approach for the Discovery of Potential Clinically Actionable Diagnostic and Prognostic Biomarkers in Colorectal Cancer
Source: Biomedicines. 2025 Jul 7;13(7):1651. doi: 10.3390/biomedicines13071651 (PMC12292145; doi:10.3390/biomedicines13071651)
Supplement: Supplementary file 1 [file biomedicines-13-01651-s001.zip › MARK-HICKS-BIOMED-CRC-SFIGS-SUBMITTED-PAPER-24MAY2025.pdf]

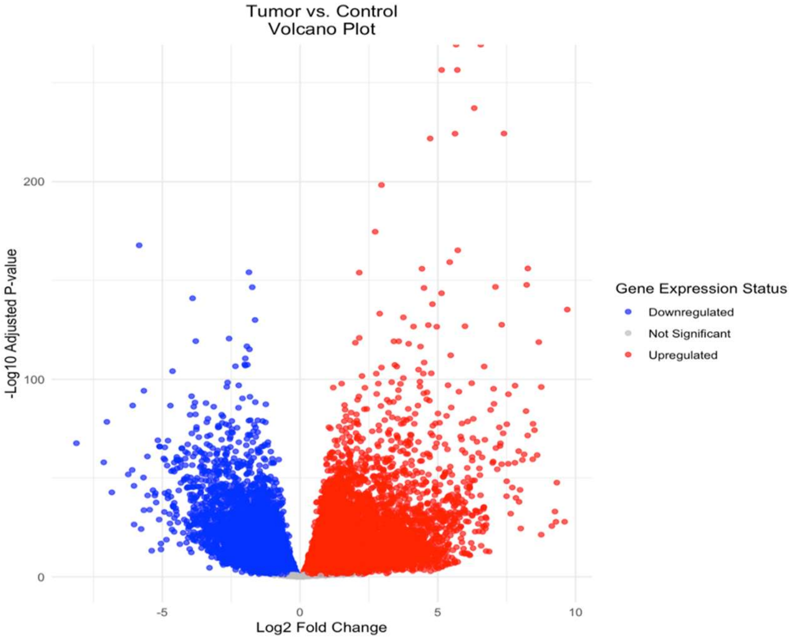

Supplementary Figure SF1: Volcano plot of significantly differentially expressed somatic mutated genes resulting from comparing gene expression values between tumor and control samples. Points colored red are significantly upregulated, points colored blue are significantly downregulated, and points colored grey are not significantly differentially expressed.

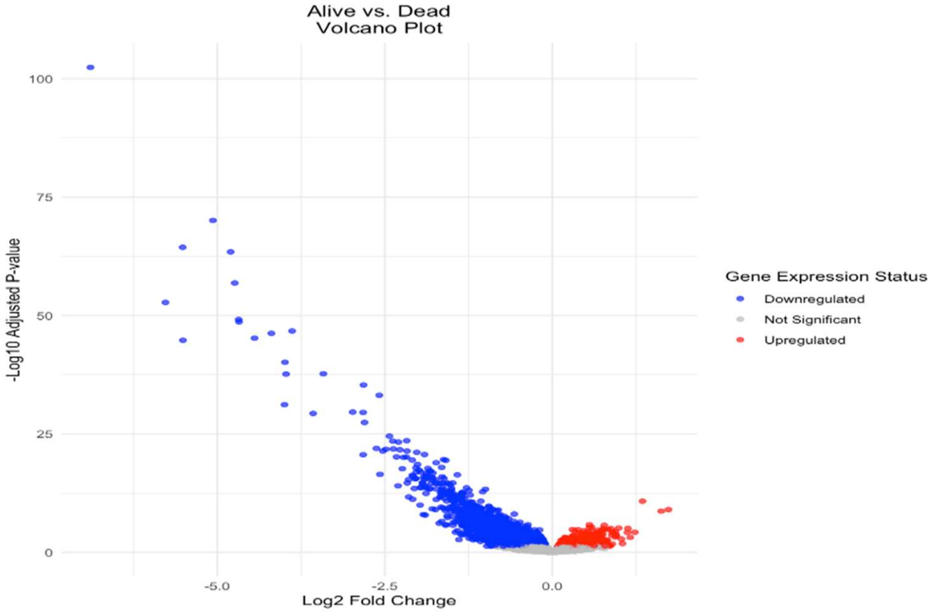

Supplementary Figure SF2: Volcano plot of significantly differentially expressed and somatic mutated genes resulting from comparing gene expression values between dead and alive samples. Points highlighted in red are significantly upregulated, points highlighted in blue are significantly downregulated, and points highlighted in grey are not significant.
